# Treatment of Produced Water Using a Pilot-Scale Advanced Electrochemical Oxidation Unit

**DOI:** 10.3390/molecules30061272

**Published:** 2025-03-12

**Authors:** Bassam Tawabini, Abdullah Basaleh

**Affiliations:** 1 Department of Geosciences, King Fahd University of Petroleum and Minerals (KFUPM), Dhahran 31262, Saudi Arabia; bassamst@kfupm.edu.sa; 2 IRC Center for Membrane and Water Security, King Fahd University of Petroleum and Minerals (KFUPM), Dhahran 31262, Saudi Arabia

**Keywords:** advanced electrooxidation, produced water, total organic carbon TOC, BDD, RSM

## Abstract

The main goal of this study is to optimize the treatment of produced water (PW) using a pilot-scale advanced electrochemical oxidation unit. The electro-cell is outfitted with a boron-doped diamond BDD anode and gas diffusion (GDE) cathode. Synthetic PW was prepared in the laboratory following a protocol designed to closely replicate the characteristics of real PW. The PW used in this study had a total dissolved solids (TDS) concentration of 16,000 mg/L and a total organic carbon (TOC) concentration of 250 mg/L. The effect of various electrooxidation parameters on the reduction in TOC was investigated including pH (2–12), electric current (I) (50–200 mA/cm^2^), and airflow rate (0–4 NL/min). Response surface method RSM with a Box–Behnken design at a confidence level of 95 percent was employed to analyze the impact of the above factors, with TOC removal used as a response variable. The results revealed that the TOC level decreased by 84% from 250 to 40 mg/L in 4 h, current density of 200 mA/cm^2^, pH of 12, and airflow rate 2 (NL/min). The investigation verified the influential role of diverse operational factors in the treatment process. RSM showed that reducing the airflow rate and increasing pH levels and electric current significantly enhanced the TOC removal. The obtained results demonstrated profound TOC removal, confirming the substantial potential of treating PW using the electrochemical method.

## 1. Introduction

Produced water (PW) is the primary waste source generated by the oil and gas sector, resulting from both onshore and offshore crude oil and natural gas production. Additionally, PW is generated during the extraction of natural gas from subterranean storage tanks [1]. Other types of wastewater are formed during oil and gas exploration, including injected water, condensed water, and residues of various chemicals utilized, with PW being the most common [2,3]. Globally, the daily production of PW is estimated at 250 million barrels compared to 80 million barrels of oil. As a result, the water–oil ratio is approximately 3:1, implying a 75% water reduction [4,5]. Oil fields account for more than 60% of all the PW generated globally [6]. In 2020, the global volume of PW was estimated to be around 340 billion barrels [7].

The PW contains various pollutants including hydrocarbons, heavy metals, suspended solids, and chemical additives such as surfactants [8]. These contaminants can cause significant environmental disruption, impacting aquatic ecosystems and food chains, and posing risk to human health [9]. For example, hydrocarbons are toxic to several marine and terrestrial species, leading to reduced biodiversity and altered ecosystem dynamics [10]. Heavy metals can bioaccumulate in the food chain [11], posing a risk to predators including humans who consume contaminated food. Moreover, the infiltration of these pollutants into groundwater sources can lead to long-term environmental and public health concerns including carcinogenic effects and other health issues [12]. Moreover, PW is hypersaline, with salinity approximately ten times that of seawater [13], releasing such saline water into the environment can disrupt the ecosystem and cause mass mortality of flora and fauna [14].

Therefore, effective treatment methods for PW and oily wastewater should be developed in order to eliminate or reduce contamination. PW is conventionally treated with a variety of physical, chemical, and biological methods. On offshore platforms, small physical and chemical systems are utilized due to space constraints. However, the current technologies are unable to remove dissolved species and small suspended oil particles. Many problems have been reported when treating PW using conventional methods. These include the phase transfer of pollutants, high cost, use of toxic additives, harmful by-products, sludge production, low efficiency, and large space requirements [15,16]. Moreover, in biological treatment, the elevated levels of salinity and other harmful contaminants in PW shock microorganisms hinder their degradation ability [17,18]. To treat high salinity wastewater biologically, halophiles—microorganisms that love salt and have slow growth rates and long retention times—are needed. A detailed review of various treatment methods for PW, including their advantages and disadvantages, is discussed by Amakiri et al. [19].

Due to these disadvantages, the treatment of PW using conventional methods is ineffective and to comply with strict waste discharge regulations, further research on technology optimization for PW treatment is needed [18]. Advanced electrochemical technologies offer a promising approach for treating wastewater comprising organic and/or inorganic compounds. Recently, interest in electrochemical wastewater treatment has grown due to its potential applications and benefits. The growing adaption of Electrochemical technologies is driving advancements in water treatment worldwide [20,21]. Several studies have reported the effectiveness of electrochemical treatments in removing persistent pollutants from various effluent compositions [22]. Electrochemical technologies are environmentally friendly because the principal species involved are oxidant agents and electrons produced in situ during the electrooxidation process. Additional advantages include the use of simple equipment, easy mobility, resilience, flexibility, and easy automation [23]. The process of electrooxidation EO, or anodic oxidation, is widely used in industrial wastewater treatment and is considered a competent treatment method. It falls within the realm of the advanced oxidation process (AOP). The popularity of EO has recently increased because of its simple installation method and effectiveness in treating hazardous and persistent organic pollutants that are normally difficult to decompose using conventional methods. The basic configuration consists of an anode and a cathode connected to a power supply. Exposure to both energy input and an adequate supporting electrolyte promotes the effective generation of strong oxidizing species. These species then interact with pollutants, promoting their breakdown. Persistent chemicals undergo a transformation process into intermediate reactions as a result of complete mineralization, which is eventually converted to water and carbon dioxide [24]. Additionally, since reactive species are produced at the anodic interface, no chemical additions are needed [25]. Aromatics, pesticides, medicines, and dyes are only a few of the toxic and non-biodegradable contaminants that EO has been used to treat. The degradation efficiencies of EO are directly impacted by the anode material. Boron-doped diamond (BDD) electrodes provide excellent corrosion resistance, even in harsh conditions, conductivity, and chemical stability. BDD has a remarkably broad potential window (2.3 V vs. SHE) compared to all the other electrodes, resulting in significant current efficiency and lower energy consumption [26]. Therefore, BDD is the ideal electrode for the full mineralization of organics.

Numerous studies have been conducted to investigate the treatment of produced water (PW) using electrooxidation methods. For instance, Souza and Ruotolo [27] studied the electrochemical treatment of oil refinery effluent containing phenolic compounds using (BDD) anodes. They investigated the effect of flow rate, current density, oxidation kinetics, current efficiency (CE), and energy consumption (EC). Their findings indicated that the BDD anode is excellent in oxidizing phenolic chemicals in effluent, with improvement in mass transfer rates and reduction in energy usage. Similarly, Gargouri et al. [28] used bench-scale experiments at varying current densities to explore the removal of hydrocarbons from produced water using PbO_2_ and BDD electrodes. They reported that BDD outperformed PbO_2_ in terms of COD removal, with a maximum removal of 96% versus 85% for PbO_2_. BDD’s increased COD removal efficiency was attributable to its higher oxidizing rate. The maximum removal efficiency was achieved in 7 and 11 h for PbO_2_ and BDD, respectively. Rocha et al. [29] investigated the electrochemical treatment of petrochemical industry PW using BDD and platinum supported on Ti (Ti/Pt) electrodes. It was found that 98% of COD removal was achieved using BDD and the COD removal rate rises with increasing applied current (from 15 to 60 mA cm^2^). In contrast, the Pt electrode resulted in approximately 50% COD removal at 30 mA/cm^2^ of current density, and applying 60 mA/cm^2^ resulted in 80% COD removal. Although energy consumption and process time can make anodic oxidation less effective for the complete treatment of petrochemical wastewater, it may serve as a viable pretreatment method to reduce the overall treatment cost and time. Abou-Taleb et al. [30] investigated phenol removal from petroleum refinery wastewater using a pilot-scale EO process, with a cylindrical graphite electrode as an anode and a stainless steel electrode as a cathode, optimizing variables such as initial concentration, time, and current. They achieved complete phenol degradation, while COD and BOD removal was 50–60%. The low removal of organic matter was attributed to the short time of the experiment, while organic matter removal using EO requires a long time. Abdulgani et al. [31] investigated the removal of COD from PW using a dimensionally stable anode (DSA) of Ti/IrO_2_ in a lab-scale system. They examined the effect of current density on COD removal and found that a maximum removal efficiency of 79% was achieved at a current density of 30 mA/cm^2^. Moreover, they studied the effect of the high sulfate and chloride concentrations in the effluent as oxidizing agents. Their findings showed that sulfate played an insignificant role, whereas chloride had a significant effect on the EO process. Yaqub et al. [32] conducted a laboratory-scale EO treatment for the removal of hydrocarbons from produced water using a DSA Ti/IrO_2_ electrode as the anode and a Ti plate as the cathode. They optimized various operational factors, including current density, contact time, and pH, employing the response surface methodology RSM and Box–Behnken design for the optimization process. At the optimized conditions (pH 3, 3.7 h, and 9 mA/cm^2^), they achieved a maximum hydrocarbon removal efficiency of 95%.

Many studies have investigated electrooxidation methods for treating different types of contaminants using BDD anodes at the pilot scale. For example, Tawabini et al. [33] investigated the removal of BTEX compounds from high-salinity waters using a pilot-scale electrochemical system equipped with a BDD anode and carbon-PTFE cathode. They optimized operational parameters including feed water flow rate, BTEX concentration, and current density using RSM with a face-centered design. Their findings showed that electric current had a significant positive impact on the electrooxidation process, while feed water flow rate and BTEX concentration exhibited significant negative impacts. The study achieved BTEX removal efficiency exceeding 80% within 120 min. In a related study, Tawabini et al. [34] employed the same pilot-scale ETS to investigate phenol mineralization in brine solutions. They studied the effect of operational parameters on phenol mineralization including pH, current density, feed water flow rate, airflow rate, and Fe^2+^. Their findings showed that the complete mineralization of phenol was achieved within 30 min. The study indicated that airflow rate and Fe^2+^ dosage had an insignificant role in phenol mineralization, while electric current had the most significant impact on the process. Moreover, Dos Santos et al. [35] explored petrochemical effluent treatment utilizing a pilot-scale system with a BDD anode. Their research demonstrated a tremendous COD reduction and energy consumption, demonstrating the feasibility of BDD-based electrooxidation for large-scale applications.

While several studies have investigated wastewater treatment using various electrooxidation methods, research on PW treatment using pilot-scale electrochemical systems with BDD as anode is limited. Therefore, this study focuses on the evaluation of pilot-scale electrochemical treatment system equipped with a BDD for PW treatment. The effects of various factors such as pH, time, current density, and airflow have been evaluated. The findings aim to evaluate the possible applicability and efficacy of BDD in large-scale treatment systems.

## 2. Materials and Methods

### 2.1. Reagents

Sodium chloride (NaCl), sodium carbonate (Na_2_CO_3_), sodium sulfate (Na_2_SO_4_), calcium chloride CaCl_2_, magnesium chloride MgCl_2_, Barium chloride BaCl_2_, strontium Nitrate Sr (NO_3_)_2_, and phenol were purchased from Sigma Aldrich (St. Louis, MO, USA) with 99% purity. Sodium dodecyl sulfate SDS (NaC_12_H_25_SO_4_) and phenol (C_6_H_6_O) at 99% purity were purchased from Thermo Fisher Scientific (Waltham, MA, USA). Three types of crude oil samples with different densities were obtained from an oil service company in the region.

### 2.2. Synthesis of Produced Water (PW)

The PW was synthesized according to the protocol proposed by Dardor et al. [36] with some modifications. A set of successive procedures are used to prepare the synthetic PW. Synthetic brine and phenol are first prepared by dissolving the various salts shown in Table 1 in one liter of deionized water. After that, 1000 mL of this brine is transferred to a 2 L beaker, and 150 mg of sodium dodecyl sulfate (SDS) is added to a 730 mg mixture of the 3 types of crude oil as shown in Table 2 to obtain a 5:1 oil–surfactant weight ratio. The solution was agitated for 30 min. at 1000 rpm. Then, the solution was sonicated for an additional 30 min to stabilize the emulsion. The mixture is then transferred to a separatory funnel and allowed to settle for 4 h to enable the separation of any free oil layer. Subsequently, the aqueous layer is transferred to a glass bottle, and the volume is adjusted to 2.5 L, preparing it for treatment in the pilot electrochemical treatment system.

### 2.3. Electrochemical Treatment Pilot System

**Figure 1:**
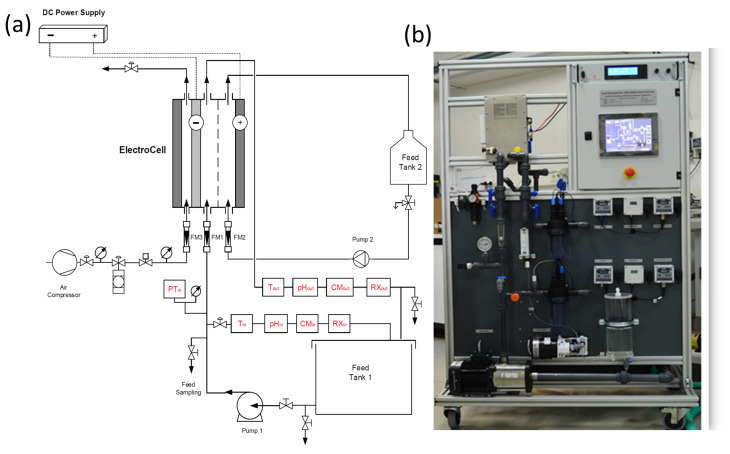
Electrochemical treatment pilot system used in this study. (**a**) Schematic diagram and (**b**) photograph of the actual system.

This study utilized a laboratory-scale pilot electrochemical treatment system (ETS) for the treatment of PW, designed and assembled by the Center for Research and Technology—Hellas (CERTH); the ETS features an Electro MP Cell from ElectroCell Co. Tarm, Denmark, a modular plate-and-frame electrochemical cell configurable in divided or undivided cell mode. The cathode comprises a carbon-PTFE GDE, and the anode is a BDD electrode, both with an effective area of 0.01 m^2^. A constant current power supply from Delta Electronika (SM 3300-series, Zierikzee, The Netherlands) with a maximum current of 22 A and a voltage of 70 V facilitated the tests. Operating parameters like conductivity, pH, temperature, pressure, and oxidation–reduction potential (ORP) were monitored using sensors at the ETS unit’s input and output. A Supervisory Control and Data Acquisition (SCADA) system governed the pilot plant via a PLC unit, incorporating a Human–Machine Interface (HMI) touch screen and various expansion electronic components. The ETS pilot plant schematic diagram (Figure 1) is composed of an electrochemical cell (plate-and-frame), a compressed air system, a DC power source, a horizontal multistage pump, an inlet tank, and various sensors measuring operational variables. The air valve from the lab air pipeline fed air to the cathode (GDE), while a regulated air system with valves, a flow meter, a relief valve, and a pressure gauge maintain pressure and airflow. To circulate the produced water (2.5 L volume) to the cell, an inverter-controlled horizontal multistage pump with a maximum flow rate of 2 m^3^/h at a head of 50 m was employed, with the liquid flow rate monitored by a float-type flow meter.

### 2.4. Experimental Procedure

The system was flushed with water prior to initiating the treatment of PW in the pilot ETS. Following that, the system was entirely emptied in order to put the PW sample. This step was repeated after each experiment to ensure the consistency of the results. To ensure homogenization, the feed solution was recirculated within the cell at a flow rate of 0.2 m^3^/h. After the pH and conductivity measurements in the cell’s inlet and outflow became steady (time zero of the experiment), the power supply was turned on (with the predetermined current levels), and the experiment began. After each experiment, the physicochemical properties (pH, conductivity, and TOC) of all the samples collected at different times (0, 30, 60, 90, 120, 150, 180, 210, and 240 min) were recorded. To determine the TOC in the liquid samples, initially, 5 mL of the sample was transferred into a 40 mL vial, and the volume was adjusted to 40 mL to achieve an 8× dilution. After thoroughly mixing the contents to ensure homogeneity, the sample was injected into the TOC analyzer. The following factors were adjusted in each batch experiment: current density (A/cm^2^), pH, wastewater sample circulation rate (m^3^/h), and airflow rate (NL/min), with the goal of identifying the best operational conditions.

### 2.5. Response Surface Method RSM

The RSM was used to explore the impacts of various operating parameters on TOC removal rate and energy consumption EC in order to find the best operating conditions. RSM, a statistical method based on a multivariate nonlinear model, is an excellent experimental design method that can reduce the number of experiments while developing a mathematical model that is best suited for the experimental data and optimizes the experimental conditions. In this study, the RSM Box–Benkhen Design (BBD) was applied to optimize the electrochemical process of PW. The three operating parameters: current density (C), airflow (A), and initial pH (B) were selected as independent variables, and their impacts on the TOC removal rate were studied. These parameters were adjusted at the beginning of each run. The experimental design was developed using the Design Expert software (version 12), consisting of 13 experiments each conducted once. Table 3 depicts the investigated factors and their corresponding levels, which were chosen to cover a wide range of values for each variable.

### 2.6. Analytical Methods

Analytical methods were employed to assess various parameters in the study. The TOC removal rate served as a key indicator for the degree of treatment in the PW. The TOC was measured using an Analytik Jena multi N/C 3100 TOC analyzer (Analytik Jena AG, Jena, Germany) equipped with an auto-sampler. The conductivity and pH of the produced water were evaluated using a PCD 650 conductivity meter (Eutech Instruments, Singapore). In addition, the MANTECH PC-BOD Multi-parameter analyzer (MANTECH Inc., Guelph, ON, Canada) was used to measure other parameters such as alkalinity, turbidity, and conductivity, ensuring a thorough examination of the water characteristics. The anion and cation concentrations were determined using a dual-column Metrohm 850 Professional IC (Metrohm AG, Herisau, Switzerland). UV-Vis Spectrometer (Analytik Jena SPECORD-50, Analytik Jena AG, Jena, Germany) was used to measure the absorbance of PW at a wavelength of 254 nm.

The removal efficiency of ETS for PW was calculated using Equation (1).

(1) $$TOC\;removal(\%)=\frac{C_{o}-C_{t}}{C_{t}}\times 100$$

where *C_o_* and *C_t_* are the initial and concentration at time *t* of *TOC*, respectively. The average current efficiency (% *CE*) for each treated solution at a particular time (*t*) was computed using Equation (2) [37], where *Co* and *Ct* are the initial and concentration at time *t* of *TOC* (gL^−1^), *F* is the Faraday constant 96,487 mol^−1^, *I* is the current in Amps, *V* is the volume of solution (L), and *t* is the reaction time.

(2) $$\%CE=\frac{Co-Ct}{8\times I\times t}F\times V\times 100$$

The energy consumption per unit of *TOC* mass at a particular time was calculated using Equation (3) [38], where *E cell* is the supply voltage (*V*), *t* is electrolysis time (h), Δ*TOC* is the change in *TOC* concentration (mgL^−1^), *V* is the volume (L), and *I* is the current (A).

(3) $$EC=\frac{E\;cell\times I\times t}{△TOC\times t\times V}$$

## 3. Results and Discussion

### 3.1. Characterization of the Synthetic PW

The characteristics of the synthetic produced water are shown in Table 4. The TOC value of 250 is attributed to the added crude oil and the SDS. This TOC value falls within the medium range, as real PW typically exhibits TOC values between 0 and 1500 mg/L [3]. The TDS value of 16,200 mg/L is predominantly due to sodium and calcium chloride, which dominate the ionic strength. This TDS level represents the middle range for PW, which varies between 4000 and 400,000 mg/L [39]. The elevated salinity significantly impacts the electrochemical process by enhancing the conductivity which improves ion mobility. However, extremely high salinity may cause electrode fouling, thereby reducing efficiency. The concentrations of sulfate, bromide and strontium, sodium, magnesium, and potassium are consistent with the reported values in real PW samples [18]. The initial pH of the PW is eight, aligning with the values reported in the literature [40].

**Figure 2:**
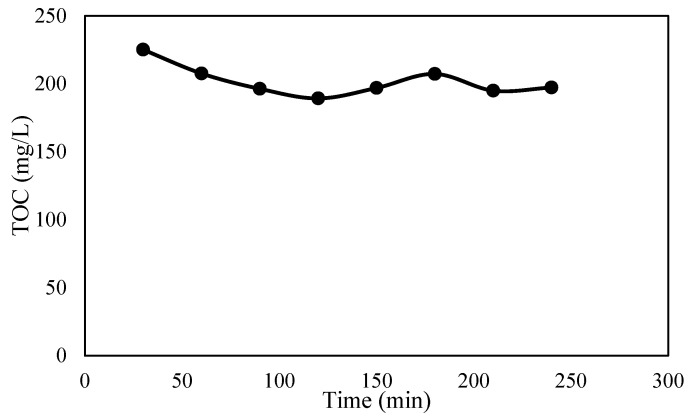
TOC levels during circulation in the pilot system with no airflow and no current.

An initial control experiment, conducted without the application of an electric field, aimed to detect any potential TOC losses resulting from adsorption or volatilization during wastewater recirculation in the pilot system. Figure 2 illustrates that after 4 h of continuous feedwater recirculation, the recorded losses were negligible. The TOC concentration remained relatively constant, with only a 13% reported loss, indicating a fairly stable system performance.

### 3.2. Effect of Electrooxidation Time

**Figure 3:**
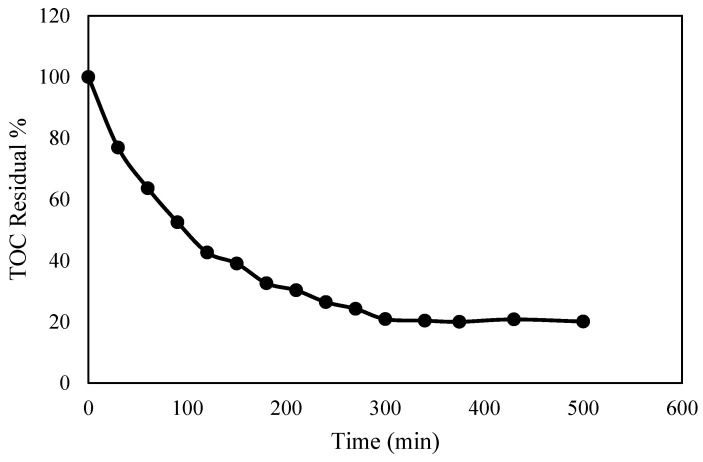
Effect of contact time on TOC removal; conditions: airflow 2 NL/min, pH 7, and I = 125 mA/cm^2^.

The treatment of the synthetic PW under moderate conditions (airflow: 2 NL/min, pH: 7, and current (I): 125 mA/cm^2^) was extended for 500 min, as shown in Figure 3. The results indicated that TOC removal increased gradually, reaching around 75% by 240 min. Beyond this point, further time extension showed an insignificant impact on TOC removal. This could be attributed to the applied current being higher than the limiting current, indicating that the electrolysis is under mass transport control. Consequently, the current efficiency is reduced due to polarization or oxygen evolution. Under these conditions, the TOC removal follows an exponential trend [41]. All the experiments in this study were conducted for 240 min, which was determined to be the optimal duration for achieving significant TOC removal.

### 3.3. Effect of Airflow Rate on TOC Removal

**Figure 4:**
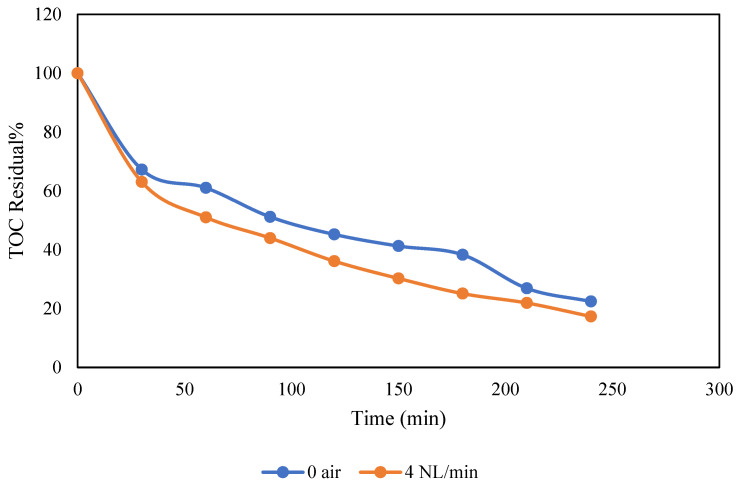
Effect of airflow in NL/min on TOC removal. Conditions: pH 12 and I of 125 mA/cm^2^.

Introducing air at the GDE cathode can enhance the electrooxidation process in undivided cells due to the electro generation of H_2_O_2_ [42]. The effect of airflow on the removal of TOC in PW was explored by passing air to the air chamber of the GDE cathode at different flow rates, specifically at 0, 2, and 4 NL/min. Figure 4 depicts the TOC removal over time under these different airflow conditions. It is evident that increasing the airflow from 0 to 4 NL/min led to a slight improvement in TOC removal. This experiment was conducted at pH 12 with a current (I) of 125 mA/cm^2^. The observed increase in TOC removal can be attributed to the enhanced airflow under these specific conditions. Increased airflow delivers additional oxygen into the system, which enhances the oxidative processes involved in TOC removal. In the presence of air, the electro generation of H_2_O_2_ occurs at the cathode, leading to the formation of more hydroxyl radicals (•OH) at the anode. This increases the degradation of organic compounds through a combined advanced oxidation/H_2_O_2_ process as shown in Equations (4) and (5) [43].

(4) $${2H}_{2}O+O_{2}{+2e}^{-}\to H_{2}O_{2}+2{OH}^{-}$$

(5) $$BDD+H_{2}O_{2}+2OH\to BDD2\left[{OH}^{o}\right]+{2H_{2}O+2e}^{-}$$

### 3.4. Effect of Initial pH on TOC Removal

**Figure 5:**
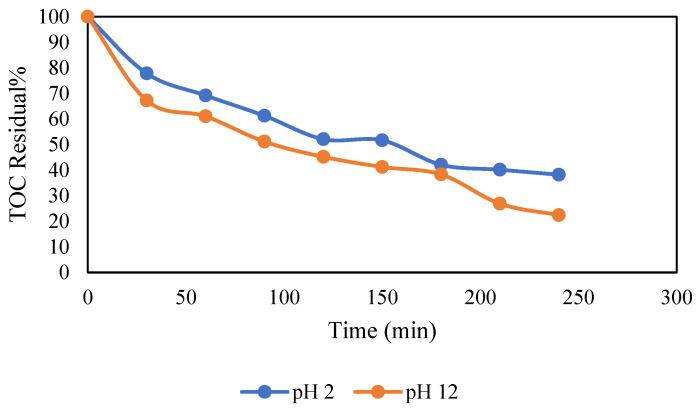
Effect of pH on TOC removal. Conditions: (a) airflow (0) and I 125 mA/cm^2^.

The effect of initial pH on TOC removal is depicted in Figure 5. The results show a clear and consistent trend wherein TOC removal increases with rising pH values ranging from 2 to 12. Specifically, over 240 min, the TOC removal increases from 60% at pH 2 to 75% at pH 12. This could be attributed to several mechanisms. The electrooxidation process at the BDD anode generates hydroxyl radicals (^•^OH), which are powerful oxidizing species. At higher pH values, the concentration of hydroxide ions increases, promoting the generation of more hydroxyl radicals. These radicals play a key role in the oxidation of PW. Moreover, at higher pH levels, the electrode surface becomes more negatively charged due to the prevalence of hydroxide ions (OH^−^) in the solution. This can enhance the adsorption of organic compounds onto the BDD anode surface. Similar results were reported by Snowdon et al. [44], where TOC removal was found to increase at higher pH levels. This was attributed to the increased evolution of oxygen, which led to the generation of more oxidative species, thereby promoting TOC degradation at BDD anodes. Another study conducted by Zeng et al. [45] reported that the TOC removal rate increased under alkaline conditions.

### 3.5. Effect of Current Density on TOC Removal

**Figure 6:**
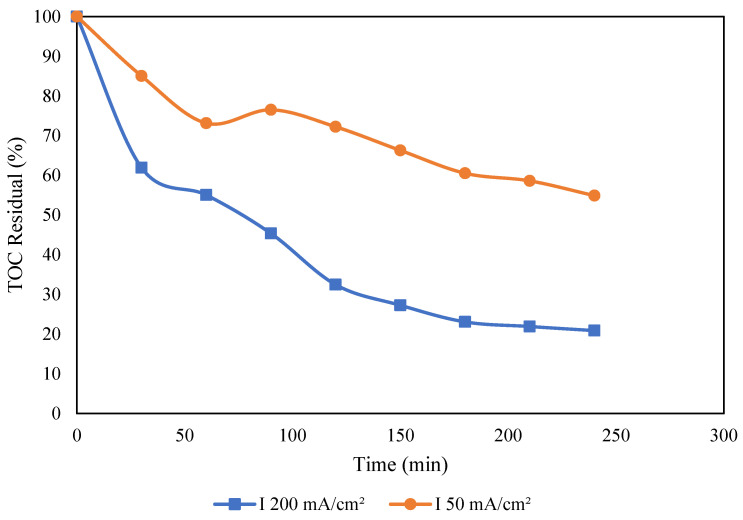
Effect of electric current (I) on TOC removal. Conditions: airflow 0 NL/min and pH 7.

Figure 6 illustrates the impact of electric current on TOC removal. The results reveal a consistent trend of increased TOC removal with rising electric current. Specifically, in Figure 6, at pH 7, there is a profound enhancement from 45 to 80 percent as the current increases from 50 to 200 mA/cm^2^. The increased electric current causes an increase in the production of reactive oxygen species (ROS), particularly hydroxyl radicals (^•^OH), which are known for their powerful oxidative capabilities [46,47]. This enhanced oxidative environment promotes the breakdown of complex organic pollutants in PW, leading to more TOC removal, as depicted in Equation (6). Furthermore, a higher current accelerates TOC removal, allowing for a more thorough breakdown of organic molecules. The chemical stability and high conductivity of the BDD anode lead to efficient electrode surface activation, which improves electrooxidation reactions. These findings are consistent with those reported in the literature [48].

(6) $$BDD+H_{2}O\to BDD\left[{OH}^{o}\right]+H^{+}+e^{-}$$

### 3.6. Statistical Analysis and Modeling

Table 5 shows the TOC removal efficiency of PW under various conditions. Statistical analysis for TOC removal was performed using the RSM (BBD) design. This analysis identified the significant factors influencing the electrooxidation process, and their respective impacts on TOC removal, with a confidence level of greater than 95%. Moreover, the energy consumption per unit mass of TOC removed under different conditions is presented in Table 5.

**Figure 7:**
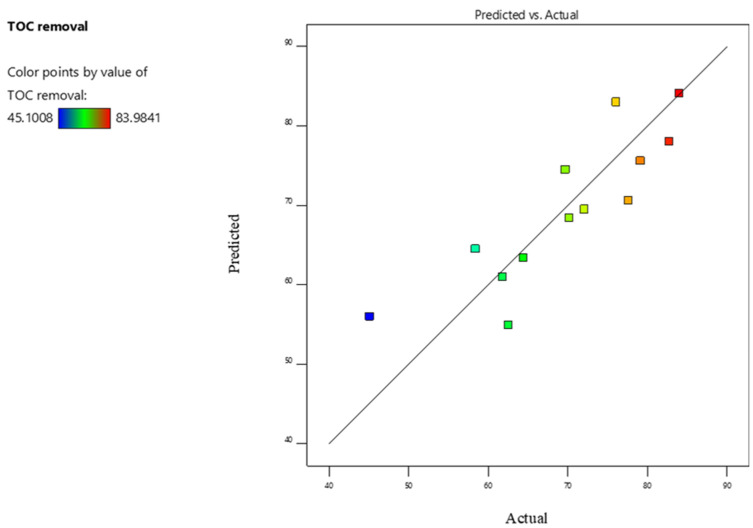
Actual vs. predicted TOC removal by ETS pilot.

ANOVA was conducted with the Design-Expert software (version 12), and certain model terms were refined to reduce bias. The ANOVA findings in Table 6 show a model F-value of 8.37, with a *p*-value less than 0.01 (0.57%), suggesting the model’s significance. The *p*-values for the model and electric current are both less than 0.05, indicating that they have a significant impact on TOC removal. In contrast, the *p*-value for airflow is 0.14, indicating statistical insignificance. While the pH *p*-value of 0.06 is slightly higher than the 0.05 threshold, it shows potential relevance, especially when assessed at the 90% confidence level. The obtained model’s adequate precision was found to be 8.094, indicating an adequate signal and confirming that the model can be used adequately to navigate the design space. The average absolute deviation (AAD) value indicates the predictive efficiency of the derived statistical models, with low AAD values indicating a highly dependable model. The obtained average absolute deviation value of 0.04% indicates that the proposed model has dependable prediction capabilities. Equation (7) presents the empirical model derived for TOC removal by the pilot ETS based on actual data. The predicted versus actual TOC removal values are plotted in Figure 7 and presented in Table 5. The obtained results demonstrated a good agreement.

(7) $$TOC\;removal\%=69.50+3.71\times airflow\left(\frac{NL}{min}\right)+4.81\times pH+9.8\times Current(\frac{mA}{{cm}^{2}})$$

### 3.7. Response Surface Plots

**Figure 8:**
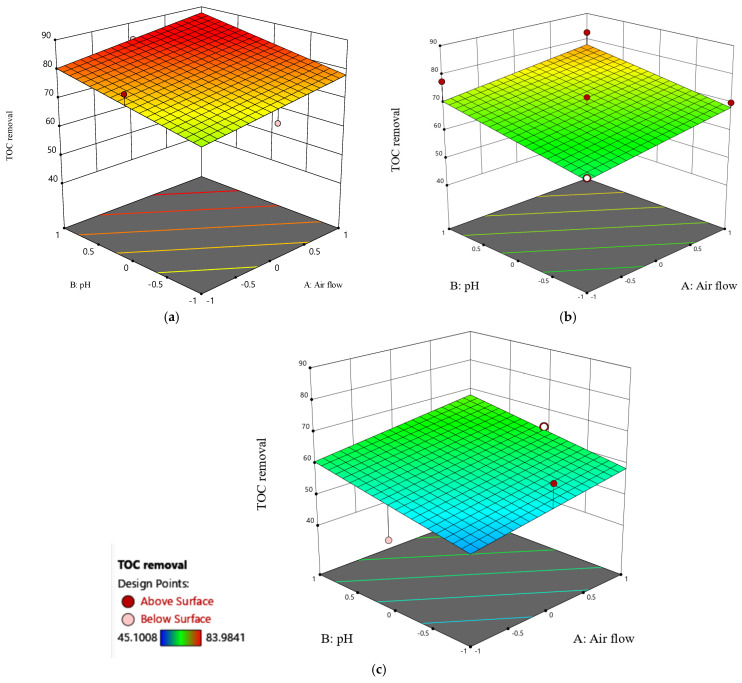
Three-dimensional surface plot of TOC removal as a function of pH and airflow under different current conditions: (**a**) 200 mA/cm^2^, (**b**) 125 mA/cm^2^, and (**c**) 50 mA/cm^2^.

Figure 8 depicts response surface contour plots and three-dimensional (3-D) graphs of TOC removal using the ETS pilot plant. The 3D response surface plot illustrating the influence of airflow and pH, along with their interaction effect, is presented in Figure 8. The subplots, denoted as Figure 8a–c, show the 3D plots at electric current levels of 200, 125, and 50 mA/cm^2^, respectively. The findings indicate a positive impact of both pH and airflow on TOC removal. As both factors increase across all the levels of electric current, there is a corresponding enhancement in TOC removal. Notably, the analysis reveals that pH has more influence than airflow on this enhancement. Furthermore, the joint increase in pH and airflow correlates with an amplified TOC removal. This observed phenomenon can be attributed to the high concentration of oxygen species, particularly hydroxyl free radicals. This synergistic effect is particularly evident when both pH and airflow are increased concurrently at a constant current of 200 mA/cm^2^ (Figure 8a). Under these conditions, TOC removal demonstrated a profound enhancement, increasing from 70 to approximately 85, confirming the significance of the combined impact of pH and airflow in improving the treatment efficiency.

**Figure 9:**
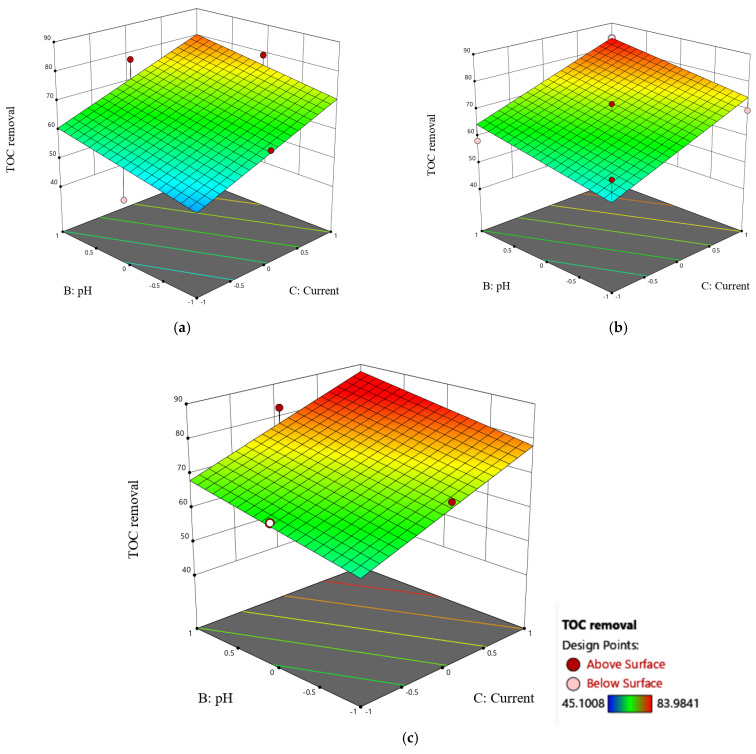
Three-dimensional surface plots of TOC removal as a function of pH and current under different airflow conditions: (**a**) 0 NL/min, (**b**) 2 NL/min, and (**c**) 4 NL/min.

**Figure 10:**
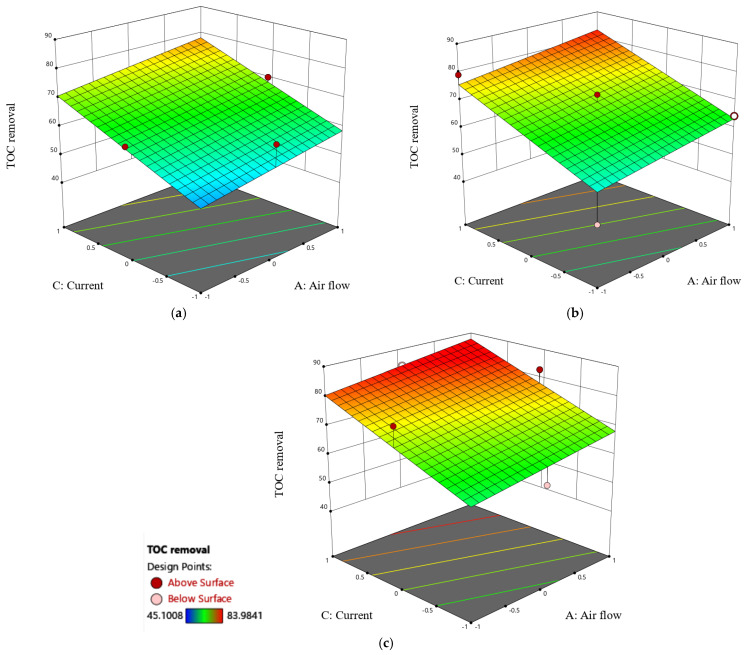
Three-dimensional surface plot of TOC removal as a function of airflow and current under different pH conditions: (**a**) pH 2 (**b**) pH 7, and (**c**) pH 12.

Figure 9 shows 3D surface plots demonstrating the effects of current, pH, and their interactions on TOC removal under different levels of airflow. Notably, both pH and current have a positive effect on TOC removal, with electric current having a stronger effect than pH. Furthermore, the simultaneous increase in pH and current yields a synergistic increase in TOC removal. This joint enhancement reflects a cooperative impact in which the combined effects of higher pH and current considerably contribute to the observed increase in TOC removal. Moreover, it was observed that the increase in airflow resulted in a marginal improvement in TOC removal. Furthermore, the 3D surface plots depicting the impact of airflow and current at various pH values are shown in Figure 10. The findings show that current has a considerable positive influence on TOC removal at different pH levels. Airflow, on the other hand, has an overall statistically insignificant positive impact on TOC removal. This observation reinforces the importance of considering the interaction of airflow, current, and pH in order to understand their combined impacts on TOC removal. The strong positive impact of current, regardless of pH fluctuations, indicates a robust contribution to the removal process, whereas the comparatively minor positive impact of airflow highlights its limited influence in these specific conditions.

### 3.8. Optimization of the Process

**Figure 11:**
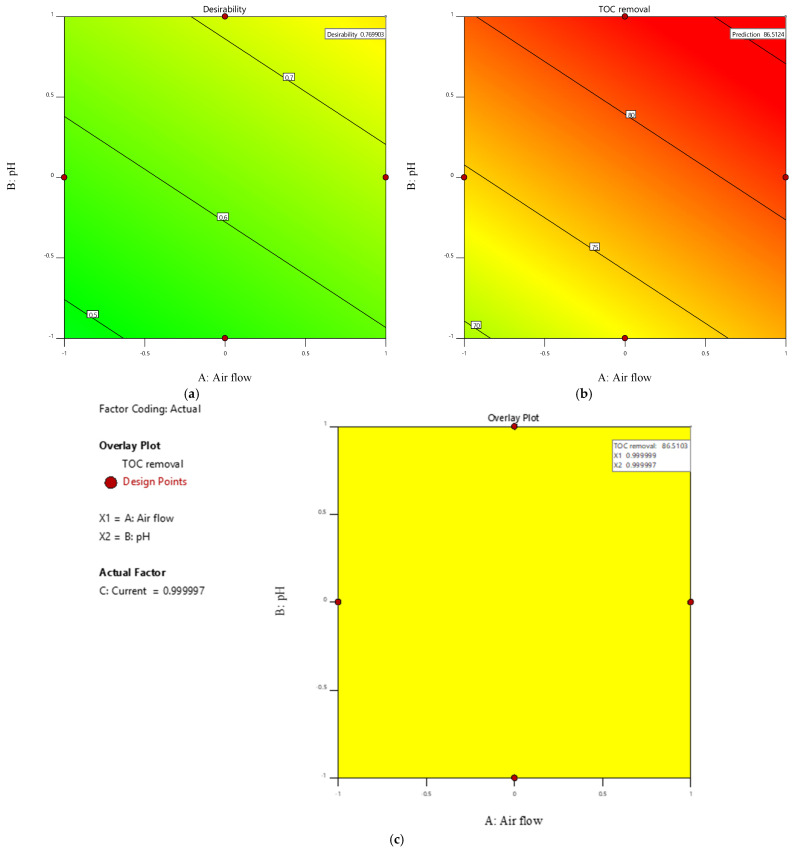
Numerical and graphical optimization for TOC removal: (**a**) desirability plot, (**b**) TOC removal response, and (**c**) overlay plot highlighting the optimal region for TOC removal.

The optimal operating conditions were identified using integrated numerical and graphical optimization tools within the Design-Expert software (version 12). Numerical optimization was configured to maximize the TOC removal efficiency as the primary response goal. The derived optimum conditions corresponded to coded variable levels of +1 for airflow (4 NL/min), pH (12), and current (200 mA/cm^2^), and yielded a predicted maximum TOC removal efficiency of 86%, with a composite desirability score of 0.77. Graphical analysis further validates these results: Figure 11a shows the desirability function delineating parameter ranges that maximize TOC removal; Figure 11b demonstrates the relation between process factors and TOC removal response, and Figure 11c presents an overlay plot, highlighting the intersection of optimal conditions and predicted TOC removal.

### 3.9. Kinetic Analysis and Current Efficiency (%)

**Figure 12:**
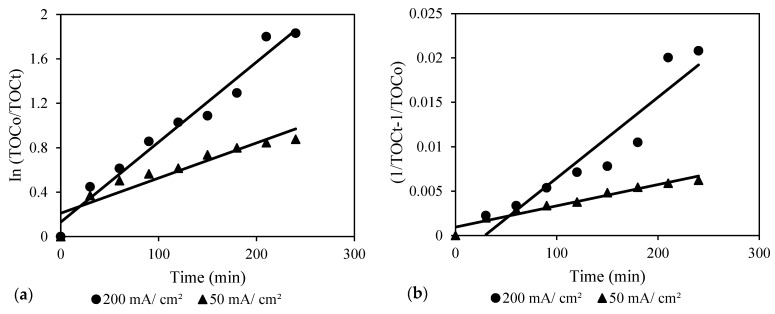

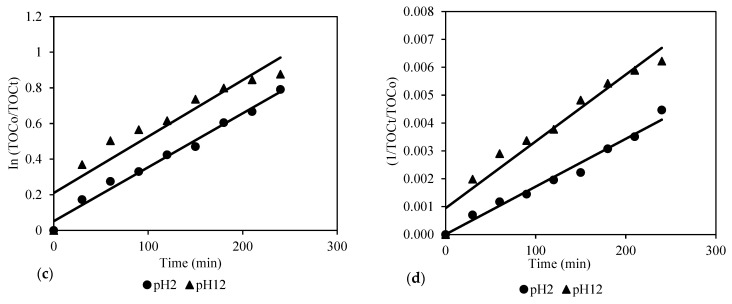
Kinetic models of the electrochemical process under different conditions: (**a**,**b**); pseudo-first- and pseudo-second-order kinetic models at different current values, pH 12, and airflow 2 NL/min; (**c**,**d**); pseudo-first- and pseudo-second-order kinetic models at different pH values, current 50 mA/cm^2^, and airflow 2 NL/min.

The experimental kinetics were evaluated where the obtained results at different conditions were fitted to the pseudo-first- and pseudo-second-order kinetic models using Equations (8) and (9), respectively. The linear plots of the obtained results with respect to time as per equations are presented in Figure 12, and the model parameters are depicted in Table 7.

(8) $$In\frac{Co}{Ct}=k_{1}t$$

(9) $$\frac{1}{Ct}-\frac{1}{Co}=k_{2}t$$

where *k*_1_ and *k*_2_ are the rate constant of the first- and second-order models, respectively. In Figure 12a,b, it was observed that at a low current of 50 mA/cm^2^, pseudo-second order followed. However, at a high current of 200 mA/cm^2^, pseudo-first order followed. At low current levels, the limited formation of free hydroxyl radicals may limit the extent of TOC decomposition. The observation of pseudo-second-order kinetics shows that the reaction rate is proportional to both the adsorbate concentration and the availability of hydroxyl radical. Initially, there is a perceptible decrease in TOC concentration as it degrades. However, when the degradation rate reaches equilibrium, a pseudo-second-order pattern arises. At this point, the degradation is less pronounced, and the TOC content stabilizes over time. At a high current of 200 mA/cm^2^, on the other hand, a substantially higher quantity of free hydroxyl radicals is generated, resulting in a more pronounced and continuous decline of TOC over time. The continual decline in TOC concentration that does not achieve a steady state corresponds to pseudo-first-order behavior. The excess of hydroxyl radicals and the rapid breakdown processes prevent the system from achieving a steady concentration in this circumstance. As limiting variables observed at low current conditions are overcome, the reaction rate becomes predominantly dependent on TOC concentration. This result was confirmed by the kinetic results obtained at different pH values as shown in Figure 12c,d. Similar trends were observed where the pseudo-second order followed at a low pH value (pH 2) due to limited hydroxyl radical, while the pseudo-first order followed at a high pH value (pH 12) due to the excess of hydroxyl radicals.

**Figure 13:**
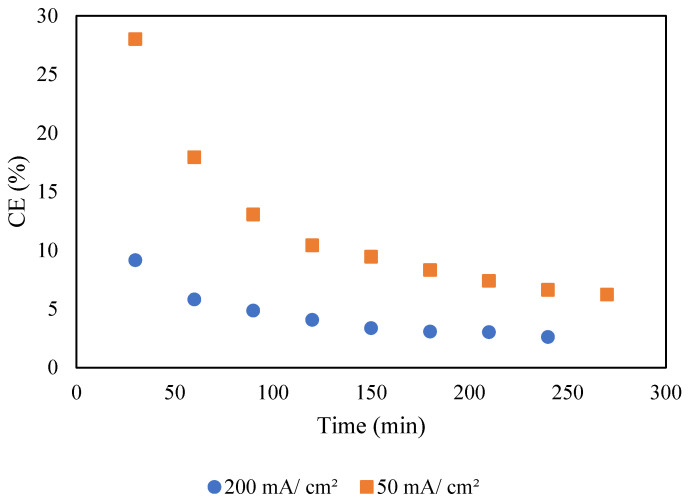
TOC removal decay with the current efficiency at different time intervals. Conditions: airflow 2 NL/min, and pH 12.

The current efficiency at different time intervals and current densities was calculated using Equation (2), and the results are depicted in Figure 13. Initially, a rapid decline in TOC was observed at the start of the process. However, it took around 4 h to achieve the highest TOC removal of 84%. This could be attributed to the mass transfer behavior. At the beginning of the process, the concentration of TOC near the electrode is high, resulting in faster removal leading to higher current efficiency. However, as the process continued, the concentration of TOC decreased, resulting in a lower transfer rate leading to a lower current efficiency. It was also observed that the current efficiency significantly decreased at higher current density. Similar findings have been reported in the literature, attributing this decrease to secondary reactions such as polarization and oxygen evolution [49,50].

## 4. Conclusions

In conclusion, this study investigated the treatment of synthetic PW using an advanced electrochemical oxidation process, focusing on a boron-doped electrode as the anode material in a pilot-scale treatment unit. The results revealed significant potential for the electrochemical oxidation process in reducing the TOC of PW, achieving a remarkable maximum removal of 84% under optimized conditions. The effect of various parameters including pH, current, and airflow was systematically explored. It was observed that higher pH levels and increased electric current substantially enhanced the TOC removal efficiency. Moreover, response surface methodology (RSM) provided valuable insights into the interaction between these parameters, highlighting the synergistic effects of these factors on removal efficiency. The statistical analysis confirmed the significance of electric current and pH on the TOC removal, while airflow was found to have an insignificant impact. Furthermore, the kinetic analysis revealed distinct patterns in TOC degradation kinetics. The pseudo-second-order kinetic model is dominant at low current, while the pseudo-first order prevails at high current levels. These findings confirm the potential of advanced electrochemical treatment methods, particularly using BDD as an anode material, in addressing the challenges posed by PW treatment. Further research should be conducted for further optimizing operational parameters to maximize the treatment efficiency.

## Figures and Tables

**Table 1:** Compositions added to prepare the brine in 1.0 L volume.

Composition	Weight (g)
Sodium Carbonate	0.05
Sodium Chloride	141.24
Sodium Sulfate	0.24
Calcium Chloride	26.81
Magnesium Chloride	5.27
Potassium Chloride	3.22
Barium Chloride	0.01
Strontium Nitrate	1.09
Phenol	0.8

**Table 2:** Properties and quantities of crude oil and surfactants used for PW synthesis.

Component	Description	Volume (mL)	Density (g/mL) 25 °C	Weight (mg)
Crude 1	Medium	0.3	0.84	252
Crude 2	Arabian Light	0.3	0.814	244
Crude 3	Condensate	0.3	0.77	231
Surfactant	SDS	-	-	150

**Table 3:** Level and value range of independent variables.

Factor	Coded Value			Real Value		
Airflow (NL/min)	−1	0	1	0	2	4
pH	−1	0	1	2	7	12
Current (mA/cm^2^)	−1	0	1	5	12.5	20

**Table 4:** Characteristics of the synthetic PW.

Parameter	Value ± SD	Parameter	Value ± SD
Chloride (Cl^−^) mg/L	6301.06 ± 150	TDS mg/L	16,200 ± 400
Sodium (Na^+^), mg/L	3776.44 ± 120	pH	8.3 ± 0.10
Calcium (Ca^2+^), mg/L	277.05 ± 20	Conductivity (μS/cm)	24,320 ± 300
Magnesium (Mg^2+^), mg/L	73.10 ± 50	TOC (mg/L)	250 ± 30
Sulfate (SO_4_^2−^), mg/L	160.94 ± 12	Turbidity (NTU)	2.49 ± 0.10
Potassium (K^+^), mg/L	61.02 ± 5.0	IC (mg/L)	6.25 ± 0.70
Bicarbonate (HCO_3_^−^) mg/L	41.40 ± 2.5	UV@254 nm	0.33 ± 0.02
Bromide (Br^−^) mg/L	3.90 ± 0.6	Temperature (°C)	23.00 ± 2.0
Strontium (Sr^2+^) mg/L	0.55 ± 0.10		

**Table 5:** TOC removal and energy consumption EC under different conditions.

Run	Coded Value			Real Value			Actual TOC Removal (%)	RSM Predicted TOC Removal (%)	EC (kWh/g [TOC]) (%)
	A: Airflow	B: pH	C: Current	A: Airflow	B: pH	**I: Current mA/cm^2^**			
1	−1	0	−1	0	7	50	45.1	50.41	0.30
2	0	1	1	2	12	200	84.0	88.73	1.71
3	0	0	0	2	7	125	72.1	69.5	0.86
4	0	−1	1	2	2	200	69.7	69.88	1.69
5	0	−1	−1	2	2	50	62.6	59.52	0.18
6	0	1	−1	2	12	50	58.4	59.89	0.38
7	1	1	0	4	12	125	82.7	77.2	1.43
8	1	−1	0	4	2	125	70.2	69.22	1.04
9	1	0	−1	4	7	50	64.4	68.99	0.43
10	−1	−1	0	0	2	125	61.8	60.17	0.90
11	−1	1	0	0	12	125	77.6	71.42	0.88
12	−1	0	1	0	7	200	79.1	81.18	1.97
13	1	0	1	4	7	200	76.0	77.43	2.39

**Table 6:** ANOVA and fit statistics for response surface linear model.

Source	Sum of Squares	df	Mean Square	F-Value	*p*-Value	
Model	1062.81	3	354.27	8.37	0.0057	significant
A-Airflow	109.95	1	109.95	2.60	0.1415	insignificant
B-pH	184.73	1	184.73	4.36	0.0663	insignificant
C-Current	768.14	1	768.14	18.15	0.0021	significant
Residual	380.93	9	42.33			
Cor Total	1443.75	12				
Std. Dev.	6.51		R^2^	0.7362		
Mean	69.50		Adjusted R^2^	0.6482		
C.V.%	9.36		AAD%	0.04		
Adeq. Precision	8.0937					

**Table 7:** Kinetic parameters of the electrochemical treatment process under different conditions.

	Pseudo-First Order		Pseudo-Second Order	
Current Density mA/cm^2^	K_1_ (1/min)	R^2^	K_2_ (1/(mg·L/min)	R^2^
200	0.0072	0.97	0.00009	0.88
50	0.0032	0.87	0.00002	0.95
pH				
12	0.003	0.98	0.00002	0.94
2	0.0032	0.88	0.00002	0.98

## Data Availability

The original contributions presented in this study are included in the article. Further inquiries can be directed to the corresponding author.
